# Ageing Effects on a Softened Bitumen by the Addition of DSA (Dodecenyl Succinic Anhydride)

**DOI:** 10.3390/polym14122437

**Published:** 2022-06-16

**Authors:** Francisco José Ortega, Antonio Abad Cuadri, Pedro Partal, Francisco Javier Navarro

**Affiliations:** Pro2TecS-Chemical Process and Product Technology Research Centre, Department of Chemical Engineeing, ETSI, Campus de “El Carmen”, University of Huelva, 21071 Huelva, Spain; francisco.ortega@diq.uhu.es (F.J.O.); antonio.cuadri@diq.uhu.es (A.A.C.); partal@uhu.es (P.P.)

**Keywords:** bitumen rejuvenation, chemical rejuvenator, rheology, viscoelasticity, viscosity, ageing

## Abstract

The softening of aged bitumen is necessary for a successful asphalt rejuvenation in road recycling operations. Thus, this study proposes a novel and successful approach by using Dodecenyl Succinic Anhydride (DSA), a reactive surfactant with a polar head capable of reacting with some polar compounds of bitumen. On this basis, this paper analyses the softening potential and ageing inhibition capability of the addition of 3 wt.% DSA before and after the application of standard laboratory ageing methods (rolling thin film oven, RTFOT and pressure aging vessel (PAV) tests). To that end, different modified bitumens were evaluated by analysing the linear viscoelastic behaviour, viscous flow properties, thin layer chromatography, Fourier transform infrared spectroscopy (FTIR), contact angle measurements and compactibility tests. The results obtained for DSA show its greater potential to soften a model bitumen, when compared to a diluent oil, through physico-chemical processes that bring about a lowering in the polarity along with the alteration of its colloidal stability. Even though ageing processes in bitumen negatively affect its softening capacity, the developed structures still retain the ability to partially compensate for the adverse hardening effects. Furthermore, DSA addition greatly enhances the binder’s wettability on a siliceous-type aggregate and favours asphalt compaction.

## 1. Introduction

During the lifespan of any pavement, even the best-constructed flexible bituminous ones will be subjected to a time-dependent deterioration of their suitable design properties, which may lead, in turn, to a significant reduction in vehicle speeds and travel comfort and affect road safety. Among others, the main factor behind this worsening is the hardening of the binder within its entire in-service temperature range as a consequence of its ageing. Even though this outcome eventually becomes clearly beneficial at high temperatures (the increase in stiffness contributes to reduce the permanent deformation of the resulting pavement), it is generally accompanied by poor performance in the low thermal range due to embrittlement of the asphaltic mixture, making the pavement more prone to undergo thermal cracking failures [1,2,3,4,5,6,7].

A number of different phenomena, such as weathering conditions, UV radiation, thermo-oxidation, etc., have been established to produce the chemical changes that bring about the referred bitumen ageing. In this sense, much effort has been devoted to the study of ageing, in order to predict the evolution of binder properties during the hot-mixing process with aggregates, in the pavement construction phase, and the progressive deterioration undergone over its lifespan [1,2,3,4]. In fact, both American Superpave and European specifications include technical requirements regarding two types of well-known ageing stages, namely short- and long-term ageing. In this sense, the so-called rolling thin film oven test (RTFOT) allows for the application of the experimental conditions to simulate hot-mix plant ageing (short-term ageing); whereas a pressure ageing vessel (PAV) is designed to provide simulated long-term aging conditions on bitumen, hence making it possible to predict the propensity of a bitumen to thermo-oxidative ageing during the pavement design life.

Based on a literature review, and leaving aside the physical hardening, considered as a reversible ageing process [1], the main ageing mechanisms that yield the largest impact on the rheological properties are related to irreversible chemical modifications [2]. The major contributors to this type of ageing are the thermo-oxidative processes and, to a minor extent, the loss of volatile components. All these processes not only bring about a notable modification on the bitumen chemical composition but also may significantly alter the structural arrangement within [4].

In terms of its chemical nature, bitumen is a complex system of different constituents, made of hydrocarbons and hetero-atoms that can be broadly classified by means of chromatographic techniques, according to their polarity, into four groups of chemical compounds: Saturates, Aromatic, Resins (which compose the maltene fraction) and Asphaltenes, making up the SARA fractions [3]. With regard to chemical changes, ageing leads to a shift in the SARA constituents toward the more polar compounds (saturates, aromatics, resins and asphaltenes), also promoting the growth of their molecular weights [4,5].

As consequence of the ageing processes, frequent maintenance and repairing operations are often required. Therefore, in order to lessen the global costs and reduce the amount of raw material consumed, recycling has increasingly become one of the most attractive alternatives for pavement rehabilitation. Nevertheless, in order for these technologies to success, the deteriorated properties of reclaimed binders, arising from ageing processes, must be restored, which can be achieved by means of the so-called rejuvenators. Rejuvenating products are aimed at restoring the physical and chemical properties of the aged binder, replenishing the volatile and dispersing oils, while also promoting adhesion [6,7,8,9,10], in order to reduce fatigue and low-temperature cracking problems [8].

To that end, most commonly, the methods applied consist in the employment of additives able to soften the aged binder by physical means, i.e., acting merely as a diluent of larger and more polar molecules. According to the literature, most rejuvenators consist of bitumen-compatible organic substances, such as lubricating and extender oils, soft bitumens, recycled motor oil, tall oils, vegetable oils (sunflower oils, waste vegetable oils, etc.), emulsions, tertiary amines, etc. [4,5,6,7,8,9,10].

Therefore, taking into account that ageing is mainly due to the chemical modification of bitumen compounds, it can then be clearly stated that none of the aforementioned additives could possibly restore the initial chemical composition but, rather, merely lower the viscosity of the aged binder by physical means [11].

A direct approach to this matter would consist in reducing oxidized groups, which is technically and economically unviable, given the complexity of the chemical process and the high cost of the reagents. Instead, a new different strategy is envisaged, focused on lowering the polarity of the bituminous compounds by attaching an aliphatic chain [12]. Since oxygen-containing chemical groups favour polar molecular interaction and the development of a more structured bitumen [13], the incorporation of a non-polar aliphatic chain would act in an opposite way, contributing to reducing the “colloidal rearrangement”, and so leading to a softening of bitumen [12,14]. This can be achieved by the addition of a reactive surfactant, in which its polar head is able to react with some polar functional groups in bitumen, whilst the aliphatic tail of such surfactant molecules projects outwards, contributing to hampering interaction with the surrounding media [12]. On the other hand, it is worth mentioning that another aspect to be taken into account is the capacity of some additives to retard bitumen ageing, playing the role of ageing inhibitors.

On this basis, this work analyses the use of dodecenyl succinic anhydride (DSA) as a reactive surfactant to modify the colloidal arrangement of the binder and, therefore, its microstructure. Specifically, the influence of short term (RTFOT) and long term (PAV) ageing has been assessed for a DSA-modified bitumen, using a light engine oil as a benchmark, for the sake of comparison. To that end, the rheological properties (viscous and viscoelastic) of bituminous binders were evaluated, along with the performance of Fourier transform infrared spectroscopy (FTIR) and contact angle measurements. Complementarily, the effect of DSA on the compactibility of a model asphaltic mixture was also investigated.

## 2. Materials and Methods

### 2.1. Materials

A neat bitumen, provided by Husky (Calgary, AB, Canada), with a penetration of 87 dmm (1/10 mm), was used as the base material to formulate modified bitumens, with a SARA composition of 3.3, 38.6, 36.1 and 22.0 wt.% for saturates, aromatics, resins and asphaltenes, respectively.

As for modification of bitumen, the following additives were used:

- Dodecenylsuccinic anhydride (DSA), supplied by Sigma Aldrich. A reactive surfactant with the empirical formula C_16_H_26_O_3_ and a molecular weight of 266.38 g/mol, exhibiting a melting point of 41–43 °C and boiling at 180–182 °C. It consists of a polar head, which corresponds to the succinic anhydride group, linked to an apolar tail, constituted by aliphatic chains.
- Succinic anhydride (SA), also supplied by Sigma Aldrich. A colourless solid characterized by the empirical formula C_4_H_4_O_3_, with a molecular weight of 100.07 g/mol, and melting and boiling points of 118–120 and 261 °C, respectively.
- Siliceous-type mineral greywacke, used for the preparation of bituminous mixtures and as a substrate in contact angle measurements. It was obtained from the “Rus/Eiffage Infraestructuras” stone quarry located in Huelva (Huelva, Spain).
- Finally, Havoline Motor Oil 10w30, manufactured by Chevron Global Lubricants (San Ramon, CA, USA) was employed as a benchmark, for the purposes of comparison.

### 2.2. Ageing Protocol

Both neat and modified bitumen were submitted to artificial accelerated ageing processes following the AASHTO PP2 standard method. With this aim, the rolling thin film oven test (RTFOT, ASTM D2872, at 163 °C for 85 min) was initially applied to simulate the short-term ageing, i.e., during processing and road construction, producing RTFOT-aged samples. Afterwards, those were further aged by using the pressurized ageing vessel (PAV, ASTM D6521, 20 h at 100 °C), which allowed reproducing the long-term ageing that takes place during the in-service life of asphaltic pavement, thus leading to PAV-aged samples.

The chemical composition of binders was determined by thin layer chromatography coupled with a flame ionization detector (TLC/FID), using an Iatroscan MK-6 analyzer (Iatron Corporation Inc., Tokyo, Japan) and utilising hexane, toluene and dichloromethane/methanol (95:5) as eluents.

### 2.3. Processing of Samples

Modified bitumens, containing 3 wt.% additive, were prepared by hot blending at the optimal processing conditions selected from previous results [12], 1 h at 150 °C, by using an IKA RW-20 (Burladingen, Germany) stirring device equipped with a four-blade turbine. Next, in order to analyse their potential for ageing inhibition, all samples were artificially aged following the procedure described in Section 2.2. Finally, in order to explore the rejuvenating capacity of the reactive surfactant, a sample of unmodified bitumen subjected to the combination of RTFOT and PAV ageing procedures (RTFOT + PAV-aged unmodified bitumen) was also modified by adding 3 wt.% DSA and stirring at 150 °C for 1 h. However, due to the small amount of material obtained after PAV, the blending process was performed on smaller aluminium cans, mixing about 50 g of aged bitumen with the corresponding amount of rejuvenator.

Complementarily, in order to analyse the sole effect of the reactive head of the DSA and to isolate the influence exerted by its aliphatic chain, a sample of unmodified PAV-aged bitumen was also blended with succinic anhydride (SA) at the same molar concentration as the DSA-modified samples.

Finally, after processing, all modified bitumens were cooled down and stored in a freezer at −20 °C before testing. This protocol allows the preservation of the microstructure until tests are subsequently conducted.

### 2.4. Tests and Measurements

#### 2.4.1. Rheological Measurements

Small-amplitude oscillatory shear measurements were performed in a control strain rheometer (ARES-G2 rheometer, TA Instruments, New Castle, DE, USA) in combination with a forced convection oven, able to control temperature with an accuracy of ±0.05 °C. This device is equipped with rectangular torsion fixtures, for low-temperature measurements, and parallel plate geometries.

Thus, depending on the testing temperature the following geometries were employed:

- For low temperatures (below 30 °C): Rectangular torsion bars with dimensions of 25.5 ± 0.1 × 12 × 2.65 mm. In order to obtain them, molten bituminous samples were poured into custom moulds and preheated at 150 °C for 5 min [12].
- For intermediate–high temperatures (from 30 to 90 °C): Smooth plate–plate geometry with a diameter of 25 mm.

Two different kinds of oscillatory tests were carried out on bituminous binders:

(a) Temperature sweep tests in oscillatory shear, from −35 to 30 °C with torsion bars and from 30 to 100 °C using parallel plate geometries, applying a continuous heating ramp of 1 °C/min, at a frequency of 10 rad/s and strains ≤ 1% within the linear viscoelastic range.
(b) Frequency sweep tests, in accordance with AASHTO T315 [15], from 0.1 to 100 rad/s, at a selected deformation within the linear viscoelastic region, with temperatures ranging from 30 °C to 90 °C and stepping every 10 °C for each test.

Before every run, samples were tempered at the desired temperature for 30 min, and at least two replicates of each test were done. As mentioned above, all these dynamic experiments were conducted at strains within the linear viscoelastic range, previously determined by means of strain sweeps at every test temperature.

Viscous flow measurements were carried out following two different protocols, each related to a specific temperature. At 60 °C, tests were conducted in a Bohlin Dynamic Shear Rheometer II, using a 25 mm diameter plate–plate geometry and 1 mm gap in a relatively wide range of shear rates (0.001–30 s^−1^). Apparent viscosity data were collected every 2 min upon reaching steady state. On the other hand, at 135 °C, bitumen viscosity was determined according to AASHTO T316 [16] by means of a Brookfield rotational viscometer, using a spindle spinning at a rate of 20 rpm.

In order to establish the low temperature performance, creep tests were performed following AASHTO T313 [17], with a bending beam rheometer (BBR, Cannon Instruments, PA, USA). Samples were moulded into rectangular specimens (125 × 12.5 × 6.25 mm), placed in the cooling bath within device and cooled down to the selected temperature. Then, flexural creep stiffness (S) and m-value parameters were determined as a function of time.

#### 2.4.2. Fourier Transform Infrared Spectroscopy (FTIR)

Testing was carried out in transmission mode on thin-film samples by means of a Nicolet Nexus 670 spectrometer. To do so, around 0.7 g of every sample was dissolved in 25 mL THF, then a droplet was carefully placed on a NaCl disk, followed by the evaporation of the solvent to obtain a thin film of bitumen. Afterwards, FTIR absorption spectra were recorded in the 625 to 4000 cm^−1^ wavenumber intervals, with a resolution of 4 cm^−1^. All spectral data registered correspond to the mean value of 20 scans of three replicates. Additionally, in order to avoid artifacts due to differences in film thickness, all spectra were normalised with respect to the CH_2_ asymmetric stretching band [18].

#### 2.4.3. SARA Analysis

Thin layer chromatography coupled to a flame ionisation Detector (TLC/FID) was used for the separation and analysis of bitumen composition, according to the SARA fractions: saturates, aromatics, resins and asphaltene, by means of an Iatroscan MK-6 device (Iatron Corporation Inc., Tokyo, Japan). Bitumen solutions were prepared in toluene (1.0% *w*/*v*), taking 1 μL of such solutions, which was carefully spotted on the bottom part of each of ten quartz rods coated with a thin layer of silica (Chromarods) and then let to evaporate to dryness. The chromatographic separation was performed by elution, using the following diluents successively: hexane, toluene and dichloromethane/methanol (95/5). Afterwards, solvents were let to evaporate in a drying oven at 120 °C. According to this separation protocol, saturates are eluted first, followed by aromatics, and finally, by resins, while asphaltenes are not eluted, staying at the spotting point. Eventually, Chromarods are scanned lengthwise, applying an air-hydrogen flame to detect the presence of the different fractions, which are then quantified by measuring the area above a horizontal baseline, and below the data collected during each Chromarod scanning. Extra details of the procedure outlined are explained elsewhere [19].

#### 2.4.4. Contact Angle Measurements

Sessile-drop contact angle measurements were carried out with the help of a dynamic goniometer, by monitoring the speeding of a hot bitumen drop laying over a mineral substrate [20].

Apparent dynamic contact angles were obtained by capturing side-images of the drop as a function of time with a CCD camera (PixeLink PL-A741) and analysing the shape of its profile. Roughly, 25–40 μL of hot bitumen were placed, using a manual brass syringe, on the surface of a polished mineral substrate (3 mm thick slab of greywacke), previously tempered at the selected thermal conditions, and bitumen spreading was measured with the axisymmetric drop shape analysis-profile (ADSA-P).

#### 2.4.5. Compactibility Tests

In order to analyse the influence of DSA on compaction operations, a semi-dense type mixture, AC16S, was selected, given its extended application in Spain. Granulometry data for both aggregates and AC16S are shown in Table 1.

The apparent relative density and the relative density on a saturated surface–dry basis is 2.66 and 2.376 g/cm^3^, respectively. The optimal binder content was set at 4.7% (of the mix weight).

As for the tests, those were performed using a gyratory compactor (D = 100 mm), in accordance with EN 12697-31 standard, at 150 °C, with a vertical pressure of 600 kPa, a compaction angle of 0.82° and at a speed of 30 rpm, applying 250 cycles.

## 3. Results

### 3.1. Influence of DSA Addition on Fresh Bitumen

In order to assess the softening capacity of DSA, a model bitumen was modified with a total content of 3 wt.% additive (at several mixing temperatures and processing times) and compared with a selected benchmark (3 wt.% of a process oil).

For the sake of clarity, since all samples exhibited Newtonian behaviour at 60 °C, over the whole range of shear rates tested, Table 2 shows zero-shear limiting viscosities obtained from steady-state viscous flow tests. As can be observed, DSA presents a superior ability to soften bitumen, regardless of the selected processing conditions. In this sense, after 1 h of processing, temperature hardly affected the viscosity, and during even longer processing times of up to 3 h, at 150 °C, gave rise to a very slight increase in viscosity, most probably due to the so-called primary ageing, i.e., thermo-oxidative processes during processing. Therefore, according to such results, and in order to allow for a better mixing while reducing the required processing time, 150 °C and 1 h were selected as optimal conditions for processing.

Moreover, Brookfield viscosities at 135 °C, gathered in Table 2, also point out a clear drop for the samples after the addition of rejuvenator, with values clearly below the limit of 3 Pa·s, as established by AASHTO T316 [16], required for the construction of bitumen pavements. This indicates a better workability and pumping potential in the construction period, making those rejuvenators suitable for its application on reclaimed asphalt pavements.

**Figure 1:**
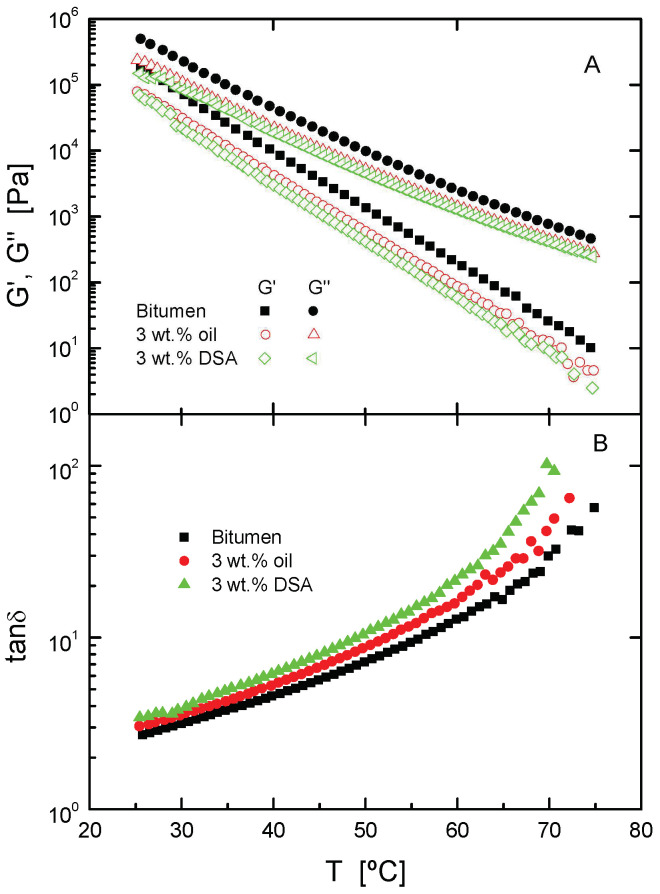
Temperature sweep tests in oscillatory shear, at 10 rad/s, for neat bitumen and unaged modified bitumen at intermediate and high temperatures. (**A**) Evolution of the storage, G′, and loss moduli, G″ and (**B**) loss tangent, tan δ, as a function of temperature.

In order to report the effect of both additives in a wider experimental range, Figure 1 presents the linear viscoelastic functions (G′, G″ and tanδ) of unaged binders versus temperature. As may be noticed, both rejuvenators bring about a noteworthy fall in both viscoelastic moduli, along with an increase in loss tangent, in the whole temperature interval.

In line with this, as shown in Table 3, the temperature at which the binder passes the Superpave rutting parameter criterion for unaged samples (|G*|/sinδ = 1 kPa) also undergoes a reduction of almost 4 °C after additivation.

**Figure 2:**
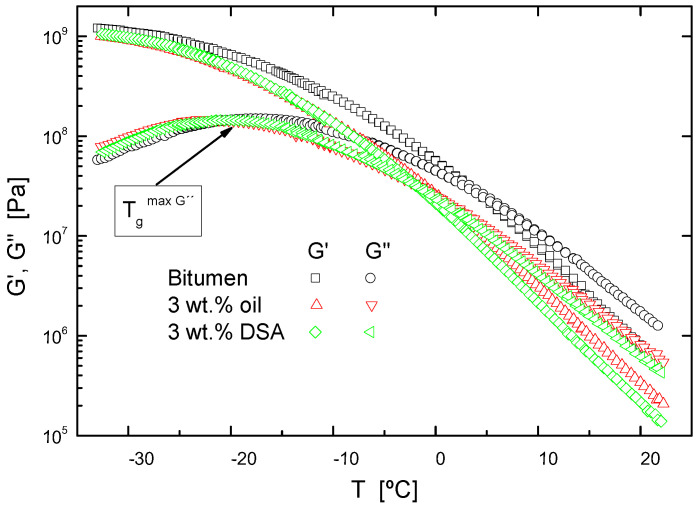
Evolution of the storage and loss moduli, from temperature sweep tests in oscillatory shear, at 10 rad/s, for neat bitumen and unaged modified bitumen, at low temperatures.

Moreover, in the low-temperature region, the so-called “mechanical” glass transition, as defined in correspondence with the temperature at which G″ reaches a maximum in the glassy zone at 10 rad/s [21,22,23], (see Figure 2), has been gathered in Table 4 for each system. According to the decrease observed of more than 5 °C in the glass transition temperature for the systems containing additives, a notable performance improvement is expected. Therefore, these results demonstrate that DSA and oil make bitumen less stiff, which is a necessary condition to ensure that the rejuvenator addition compensates for the deteriorated mechanical properties, in order to avoid cracking failures in asphalt pavement [21,22,23]. In addition, once again, Figure 1 points out the greater efficacy of DSA when softening a model bitumen, so as to mechanically rejuvenate it.

### 3.2. Ageing Effects on Neat Bitumen and Rejuvenation

**Figure 3:**
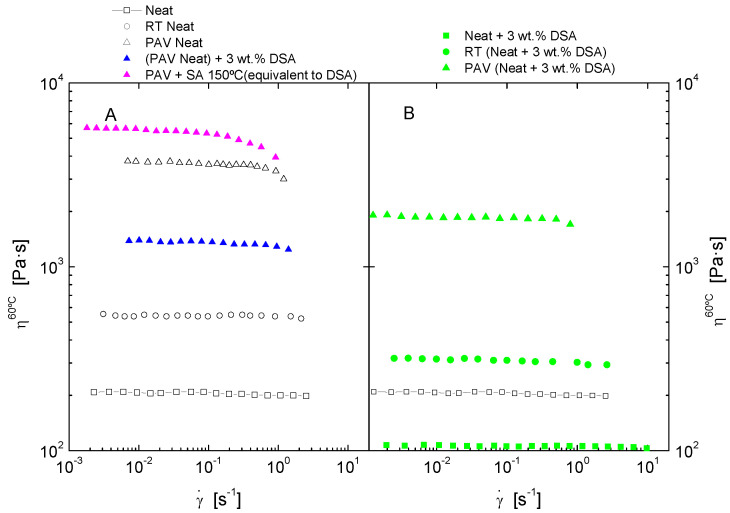
Viscous flow curves, at 60 °C, for (**A**) neat and aged bitumen by RTFOT (RT) and PAV protocols and effect of the addition of DSA and succinic anhydride (SA) on the PAV-aged sample and (**B**) Unaged bitumen modified by DSA and ageing effects on the modified bitumen.

**Figure 4:**
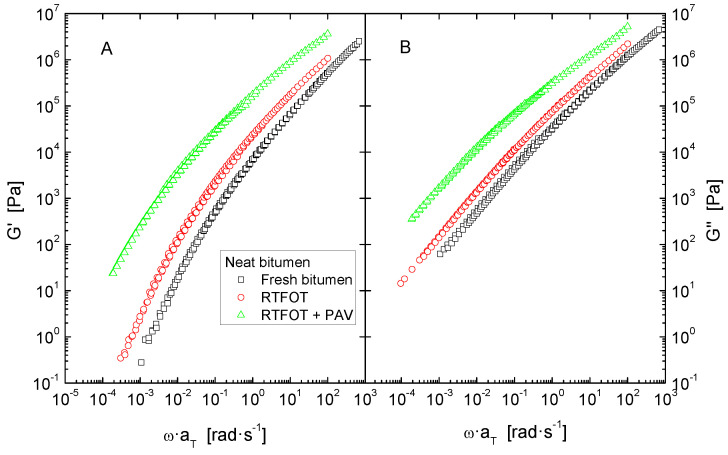
Master curves of storage (**A**) and loss moduli (**B**), at a reference temperature of 30 °C, for neat bitumen, before and after ageing by RTFOT and RTFOT + PAV.

**Figure 5:**
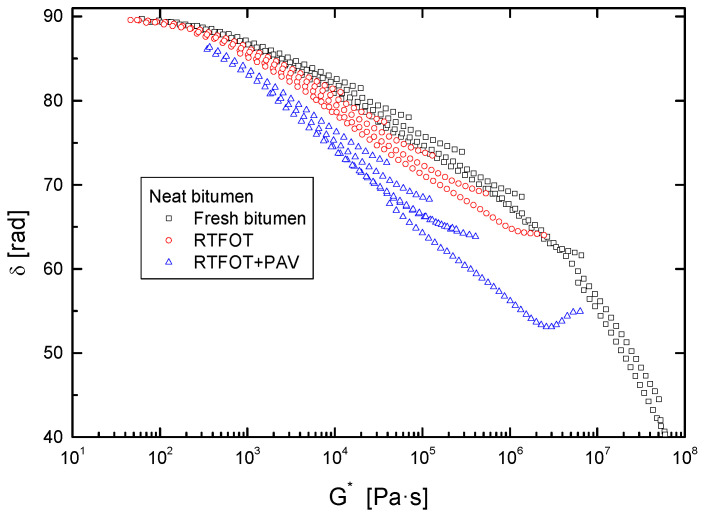
Black diagrams (phase angle, δ, versus complex shear modulus, G*) of neat bitumen, before and after ageing by RTFOT and RTFOT + PAV.

The effect of both short and long-term ageing was simulated by subjecting samples to the consecutive ageing methods RTFOT and PAV. After every ageing process, flow curves at 60 °C (Figure 3) and isothermal oscillatory linear viscoelastic measurements (Figure 4 and Figure 5) were performed on each sample.

Firstly, data from viscous flow tests at 60 °C allow the evaluation of the hardening effect at different ageing stages for both neat and DSA modified bitumen and the analysis of the ageing inhibition capacity of DSA. In this sense, Figure 3 shows that both samples undergo a remarkable increase in viscosity, especially after the PAV test. Included data of a rejuvenated PAV-aged bitumen, i.e., modified with DSA after ageing tests, can be used to determine the DSA’s real potential of rejuvenation. As may be observed, the addition of 3 wt.% DSA lowers the binder viscosity noticeably. However, due to the low additive concentration, DSA cannot completely restore the fresh bitumen properties and, thus, a much larger concentration would be required to achieve a full rejuvenation. In addition, the reported ageing evolution of DSA modified bitumen sample points out its anti-ageing capacity or ageing inhibition. On the other hand, the addition of DSA after PAV ageing results are slightly more effective than the DSA modification before ageing. This difference is attributed to the degradation/volatilization of DSA during ageing protocols, which is analysed in Section 3.4.

**Figure 6:**
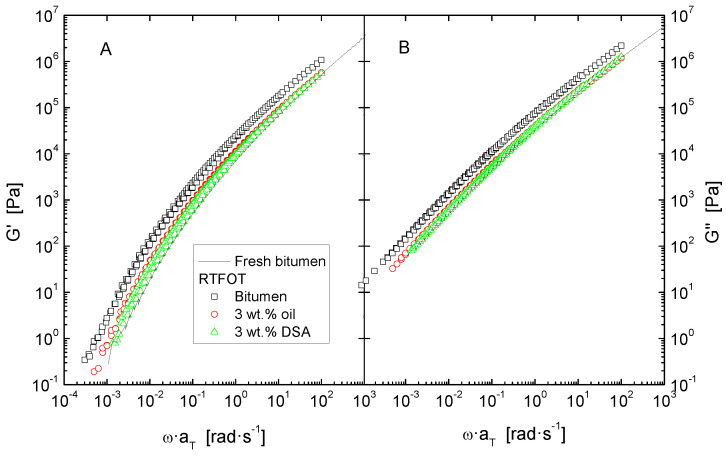
Master curves of storage (**A**) and loss moduli (**B**), at a reference temperature of 30 °C, for neat bitumen and rejuvenated bitumen after ageing by RTFOT.

**Figure 7:**
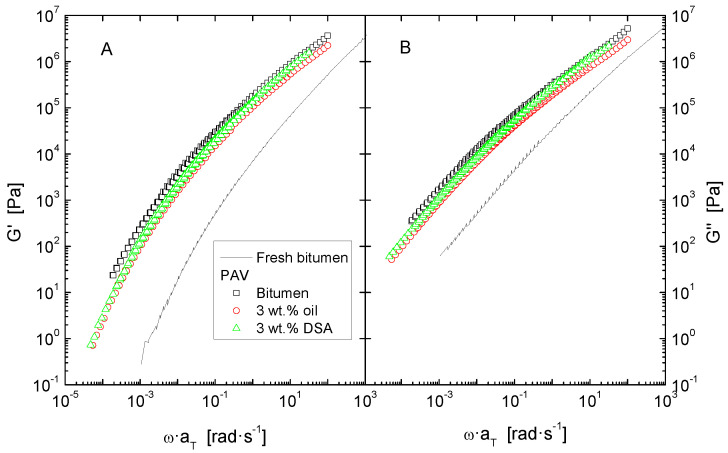
Master curves of storage (**A**) and loss moduli (**B**), at a reference temperature of 30 °C, for neat and modified bitumen after ageing by RTFOT + PAV.

Secondly, values of the dynamic material functions were used to build a master curve by applying horizontal shift-factors, a_T_, without any vertical shifting, in implementation of the time–temperature superposition principle (Figure 4, Figure 6 and Figure 7). The reference temperature was kept at 30 °C, and the shift factors follow a Williams–Landel–Ferry (WLF) dependence (Table 4):

(1) $${\mathrm{loga}}_{T}=\frac{-C_{1}\left(T-T_{\mathrm{ref}}\right)}{C_{2}+\left(T-T_{\mathrm{ref}}\right)}$$

In general, in the case of bituminous products, superposition principle does not always give rise to a smooth master curve, pointing out a deviation from a thermorheologically simple behaviour. This is not surprising, given the structural complexity and heterogeneity of bitumen, with the presence of dispersed phases which may undergo structural changes in the transition zone, as in the cases of highly crystalline bitumens (wax contents > 7 wt.%), structured bitumens with high asphaltene contents and highly polymer-modified bitumens [22].

For the sake of clarity, a Black diagram (complex modulus against phase angle) is presented in Figure 5 to avoid misleading information due to the shifting procedure, since both frequency and temperature are eliminated from the plot. These types of graphs are commonly employed not only for confirming the applicability of the time–temperature superposition principle but also for assessing changes in the resulting structure or composition of the system. As shown, Figure 5 displays the well-known pattern of conventional bitumen, characterized by a wide Newtonian viscous flow region at low G* values (where phase angle approaches 90°), followed by a steady diminution of δ [23]. Figure 5 also illustrates that ageing produces a significant influence on the rheological response, especially at high stiffness values, corresponding to low temperature and high frequency ranges. In this region, a shifting of the curve towards lower phase angles is clearly noticed, with the consequent increase in its elastic features, pointing out the aforementioned hardening effect. On the other hand, as ageing proceeds, Black curves begin to lose their smooth trends, revealing a more complex rheological behaviour. These results have been associated with modifications in the microstructure, given the compositional changes that the bituminous fractions undergo as a consequence of thermo-oxidative reactions, bringing about a greater presence of more polar molecules, as discussed later [5,24].

Furthermore, despite the features observed in Black diagrams, it is very common that storage and loss moduli curves merge into a single common master curve when plotted against reduced frequency, which can then be used for comparison (Figure 4, Figure 6 and Figure 7). In general, all systems present a similar evolution of their viscoelastic functions, characterised by a steady increase of both G′ and G″ with frequency, in correspondence with the distinctive gradual continuous transition from the glassy to the Newtonian region.

Data in Figure 4 also provide a clear insight into the effect of accelerated ageing in a wider range of frequency. Thus, as widely reported in the literature, in the whole experimental window, either ageing simulation method applied gives rise to an upward shift of both elastic and viscous curves [5,25]. It is important to underline that long term ageing (PAV) exerts the largest impact on the rheological response, especially at low frequencies (or high temperatures), where a rise of more than two orders of magnitude is evidenced for both moduli.

### 3.3. Ageing Effects on Softened Bitumen

As pointed out, DSA may effectively act as a bitumen rejuvenator and ageing inhibitor, since its addition brings about a notable softening over a wide range of temperatures, partially compensating the hardening caused by ageing (Figure 6 and Figure 7).

However, after rejuvenation, the use of the resulting binder in new mixtures requires to properly analyse its susceptibility to ageing during production and construction operations (short term) and after pavement service life (long term).

In this sense, high temperature stiffness and fatigue resistance of the aged samples were determined according to AASHTO specifications, resulting in the values included in Table 3, with the critical high temperatures for rutting after RTFOT (G*/sinδ > 2.2 kPa (AASHTO T315), and the critical fatigue temperature after PAV (G*·sinδ < 5 MPa, AASHTO T315) are clearly lower for the modified bitumens. Consequently, both rejuvenators yield a decrease in binder stiffness at either ageing stage, both at intermediate and high in-service temperatures and, therefore, give rise to a softened binder with improved fatigue strength.

The effect of accelerated ageing on rejuvenated samples in a wider temperature range is presented in Figure 6 and Figure 7. In accordance with the evolution of the elastic and viscous moduli for neat and modified bitumen after RTFOT and PAV, in both cases, the curves for the rejuvenated bitumen remain below those of the corresponding unmodified, aged bitumen. This demonstrates the benefit obtained on the thermomechanical behaviour of the former, as both oil and DSA-modified bitumen are found to be less stiff than the untreated binder after ageing. However, it is worth noting that the quantitative effect of RTFOT and PAV on the rheological properties is clearly different. In the case of short-term ageing, as depicted in Figure 6, DSA-rejuvenated bitumen presents the largest softening degree, showing similar values to the unaged bitumen. By contrast, after PAV, oil and DSA-modified samples undergo a remarkable hardening yet exhibit moduli values lower than those of the untreated PAV-aged bitumen (Figure 7).

In order to clarify this issue and to explore further changes in the rheological properties of modified bitumens with the ageing degree, a relative softening index (RSIT), at a selected temperature, T, was defined as follows:

(2) $$RSI_{T}={\left(\frac{G_{neat\;}^{*}-G_{3\%\;additive}^{*}}{G_{neat}^{*}}\right)}_{T}$$

where G* is the complex shear modulus at the selected temperature (30, 60 and 90 °C), taken from frequency sweep tests, at 10 rad/s, after performing the same ageing test. This parameter can hence be considered a measurement of the softening capability of rejuvenators, when subjected to the same ageing protocol, so that the higher the index the better the rejuvenating effectiveness.

**Figure 8:**
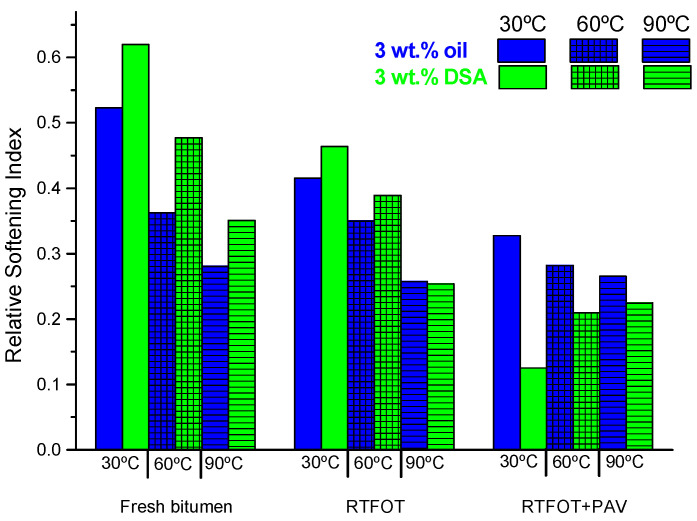
Relative Softening Index values for neat and modified bitumen after ageing by RTFOT + PAV at different testing temperatures.

In the case of the additives assessed in this work, the values obtained for this parameter are positive, as portrayed in Figure 8, pointing out the softening efficacy of both additives and their ageing inhibition capacity. By comparing the indexes at the same temperature for the unaged samples, once again, the DSA-containing binder presents better results than the oil-modified bitumen. However, as ageing proceeds, the effect of both additives progressively diminishes, reducing the differences between DSA and oil.

It is also noteworthy that, after PAV, an opposite tendency is observed, since the best result is now displayed by the oil-modified bitumen. This finding hints at some type of adverse chemical process that happens after long-term ageing as a consequence of the combined effect of pressure and time. In fact, these results seem to contradict those obtained when assessing the effect of processing of samples, as reported in Table 2, where modification due to mixing time and temperature was almost negligible. For example, RTFOT (performed at 163 °C during 85 min) yields more pronounced changes in the rheological response than samples processed at higher temperatures (180 °C after 1 h).

Nonetheless, this seemingly contradictory result may be related to the larger surface exposed to air both in RTFOT recipients and PAV pans, which tends to favour oxygen diffusion and, therefore, thermo-oxidative reactions. Consequently, oxidative processes will likely prevail over thermal ones.

Finally, BBR tests on PAV-aged samples were used to determine the low temperature properties and to characterize the cracking resistance of every binder. Table 3 illustrates the limiting temperature at which thermal cracking is expected, as established by AASHTO T313 (S(t) < 300 MPa and m-value > 0.3). In this regard, BBR results indicate that cracking failures would appear at lower temperatures for oil and DSA-modified binders. Furthermore, according to S(t) values at −12 °C and G′ values, at low temperatures, as shown in Figure 2, it can be easily deduced that binder stiffness is reduced and, therefore, the modification gives rise to better low-temperature performances.

### 3.4. Chemical Reactions and Microstructure

In general, the reported [26] results may be explained on the basis of chemical reactions of bituminous molecules with oxygen of air and with the anhydride group in DSA.

**Figure 9:**
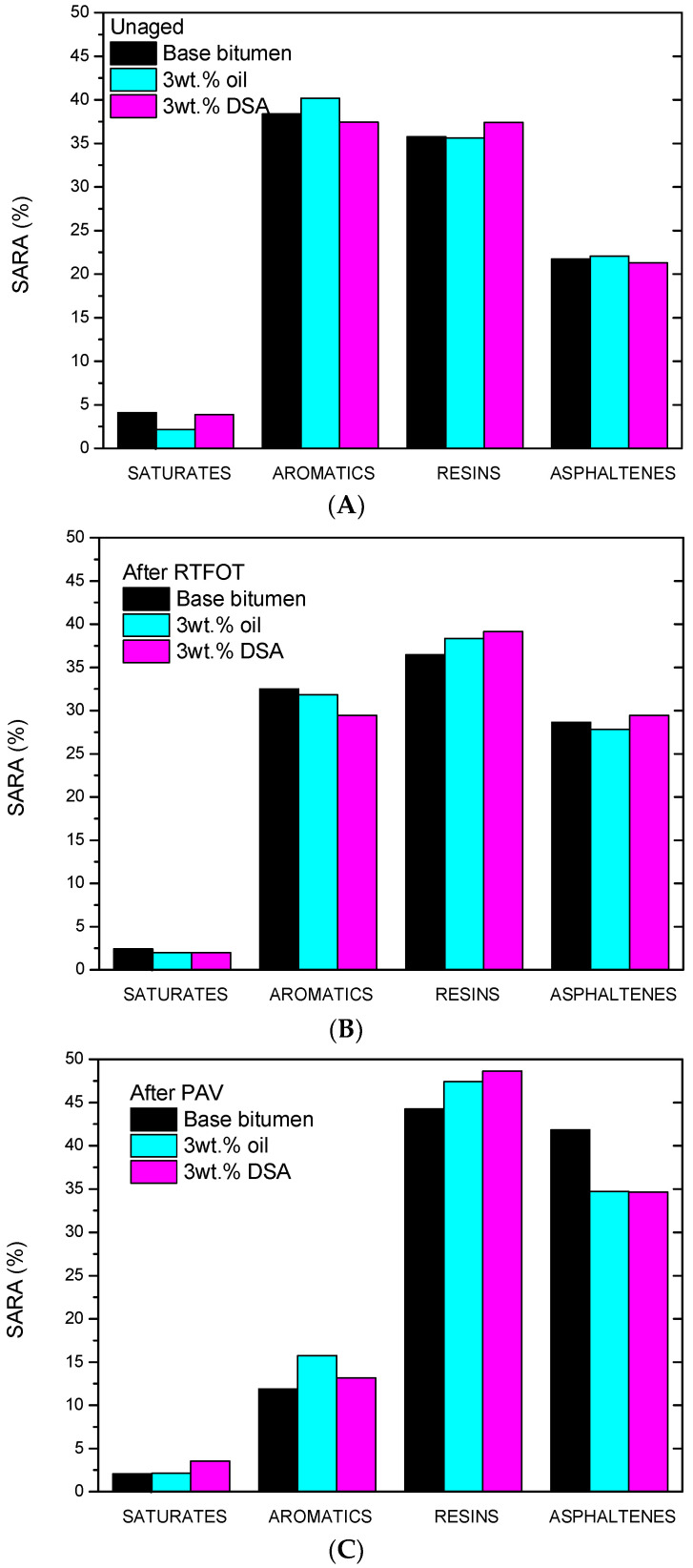
SARA composition for neat and modified bitumen (**A**) before ageing, (**B**) after RTFOT and (**C**) after RTOFT + PAV.

Firstly, SARA analysis, presented in Figure 9, reveals a great susceptibility of unmodified bitumen to ageing chemical changes. It is well known that ageing results from a combination of thermo-oxidation and polymerization reactions, along with, to a lesser extent, the evaporation of lighter components, leading to a modification as follows: aromatics → resins → asphaltenes [26]. In this case, the modification observed in SARAs fractions after RTFOT and PAV processes is consistent with the stiffening effect identified by rheological techniques. Thus, for all samples (neat and modified bitumen), as ageing proceeds, aromatics content lowers, while the concentration of resins and asphaltenes rises, especially after PAV. This is particularly remarkable for unmodified bitumen, where the asphaltene content doubles after PAV and resins undergo a notable reduction.

From a microstructural perspective, it is important to note that these chemical changes may alter the colloidal structure of bitumen and, therefore, its mechanical properties. This effect can be analysed using the so-called Gaestel colloidal index (C.I.), calculated as follows [27]:

(3) $$C.I.=\frac{\mathrm{saturates}+\mathrm{asphaltenes}}{\mathrm{resins}+\mathrm{aromatics}}$$

**Figure 10:**
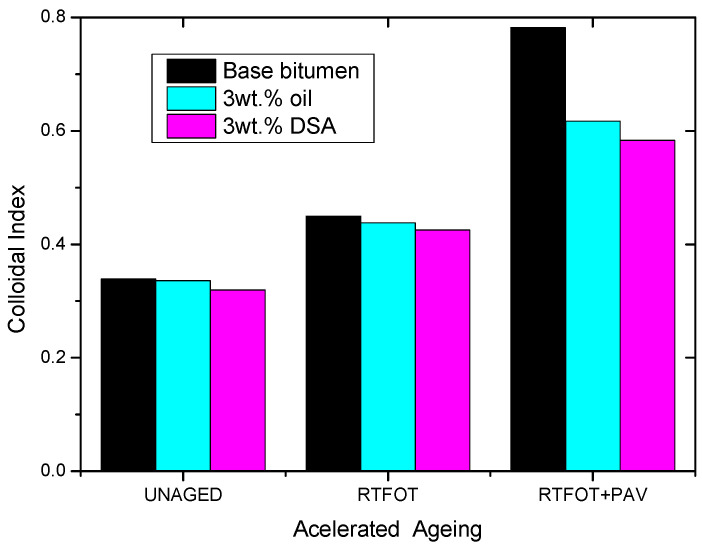
Colloidal Index values for neat and modified bitumen after RTFOT + PAV.

According to the colloidal model, the C.I. values portrayed in Figure 10 allow us to determine the evolution of the “sol-gel” character of bitumen’s microstructure with ageing, since a higher index value is related to a material exhibiting a more significant solid-like behaviour. Hence, the larger C.I. values obtained after ageing tests may explain the hardening observed at high-in service temperatures. By contrast, at any ageing stage, both additives yield a reduction in the C.I. values, which would consequently account for the observed softening/ageing inhibition effect of oil and DSA. Figure 10 also shows that differences in colloidal indexes become more intense as ageing proceeds (especially after PAV), hinting that both additives not only soften the binder but also enhance the ageing susceptibility of bitumen.

The modification of the rheological properties exerted by DSA is attributed to chemical interactions between its pendant succinic anhydride groups and some bitumen molecules, as may be deduced from FTIR spectroscopy.

**Figure 11:**
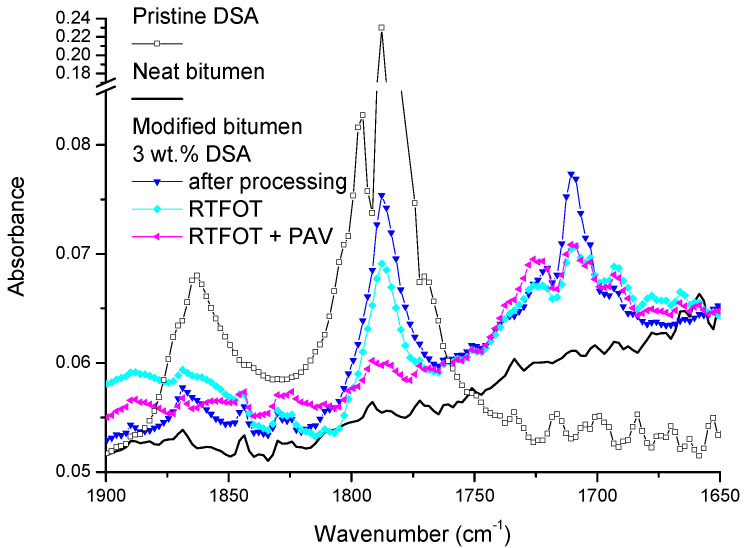
FTIR spectra for pristine DSA, neat bitumen and DSA modified bitumen after processing and after ageing by RTOFT and by RTOFT + PAV.

In this regard, Figure 11 allows for a comparison of the normalised spectra of pristine materials with DSA-modified bitumen. Firstly, data for pure DSA show the presence of two characteristic stretching bands of the carbonyl groups in the cyclic anhydride, located at 1785 and 1863 cm [18], while unmodified bitumen does not display any of these bands. After processing, the anhydride peaks undergo a clear intensity reduction in DSA-modified bitumens, attributable to ring opening reactions involving bitumen molecules. The formation of new linkages is confirmed by the appearance of a new broad absorption band in the range of 1700–1750 cm^−1^ after DSA modification. As a matter of fact, cyclic anhydrides are known to react with molecular species that contain hydroxyls or amine groups, both of them present in bitumen [28], to produce new ester and amide bonds, respectively, in addition to a pendant carboxylic acid group. Consequently, the appearance of this new IR band is partially due to the overlapping absorption of carbonyl groups from the pendant carboxylic acid and that of the carbonyl groups in esters [29]. Furthermore, the reaction of DSA with amine groups in bitumen may form new succinimide linkages [30], leading to an imide stretching absorption band within the same wavenumber range (typically at 1720 cm^−1^).

**Figure 12:**
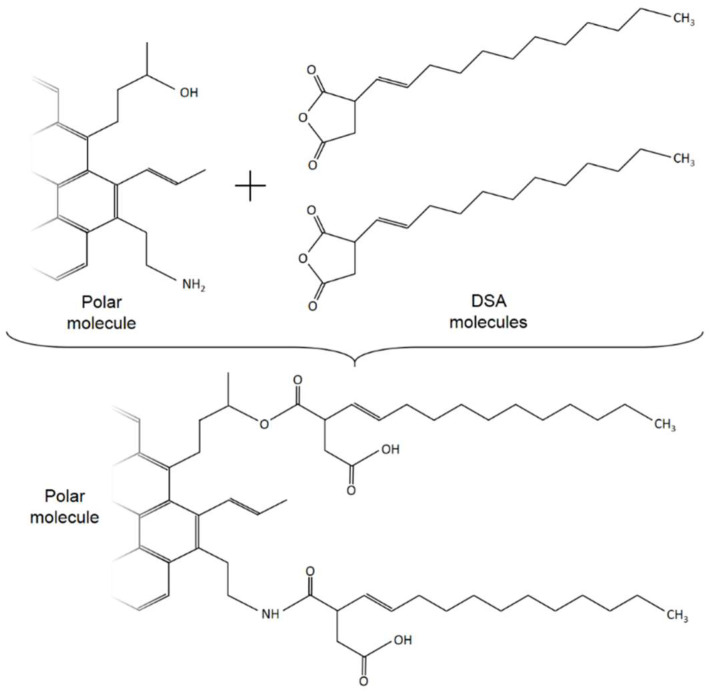
Expected chemical reactions between DSA and bitumen polar molecules.

As shown in Figure 12, such a successful succinylation of polar bitumen molecules (resins and asphaltenes) with DSA leads to the attachment of its non-polar long alkyl chain within bitumen molecular structure, resulting in the diminution of bitumen’s polarity, which eventually affects its colloidal stability, as evidenced by the drop in the values of the colloidal index (Figure 10). This issue can be better analysed through the comparison with the rheological modification caused by the addition of succinic anhydride (SA) and DSA. Thus, Figure 5 shows that SA (which corresponds to the reactive polar head of the surfactant) produces a small structuring effect on the PAV aged sample since viscosity is slightly increased, whereas DSA modification brings about a notable viscosity drop. Consequently, it can be clearly deduced that the presence of the aliphatic tail is the responsible of the reported disruptive effect that gives rise to the reported softening.

On the basis of the colloidal model, bitumen structure results from the association of asphaltene molecules into nanoaggregates, which are further combined into clusters solvated with resins, forming supramolecular micellar structures [31]. Therefore, the degree of molecular association is directly related to the bitumen’s rheological response. In this regard, the attachment of the non-polar tail of DSA to bitumen polar molecules hinders their stacking into larger structures, thus serving as a steric barrier against molecular attraction and agglomeration [32]. Consequently, DSA contributes to inhibit the grouping of asphaltenic structures, ultimately giving rise to a lower degree of microstructural complexity, which supports the reported softening [33].

Finally, Figure 11 also contributes to a deeper insight into the modification observed in the ageing tendency after DSA addition. FTIR data show that the stretching bands of the cyclic carbonyl groups in the succinic anhydride (at 1785 and 1863 cm^−1^) tend to level off after ageing tests, as a result of the further linking of unreacted DSA molecules by the action of time and temperature. This beneficial process adds to the evaporation/degradation of DSA during ageing protocols, as evidenced by the higher weight loss reported in Table 3. However, in spite of this, the band ranging from 1700 to 1750 cm^−1^, assigned to the reaction products, barely diminishes after RTFOT and PAV. This result clearly hints that a number of DSA molecules remain attached to polar bitumen compounds after ageing and, therefore, the structures developed still retain the ability to alter the colloidal stability of the binder.

### 3.5. Contact Angle and Asphalt Compactibility

The angle that the contour of a droplet of hot bitumen forms when placed on the surface of an aggregate defines the so-called contact angle, which allows the assessing of the interactions that exist between both materials at their interface, and so constitutes a method for the evaluation of bitumen’s wettability, a key factor to ensure success in mixing, lay-down and compaction operations [34]. In this sense, contact angle measurements have been widely reported in the literature as a valuable tool to estimate bitumen’s adhesion capacity, also exhibiting good correlation with usual empirical standard methods [35]. Correspondingly, strong adhesion is needed to ensure good roadway performance and durability.

Here, instead of static angle tests, dynamic contact angles were determined, as they better describe the physicochemical interactions between bitumen and aggregate when spreading at high temperature [20].

**Figure 13:**
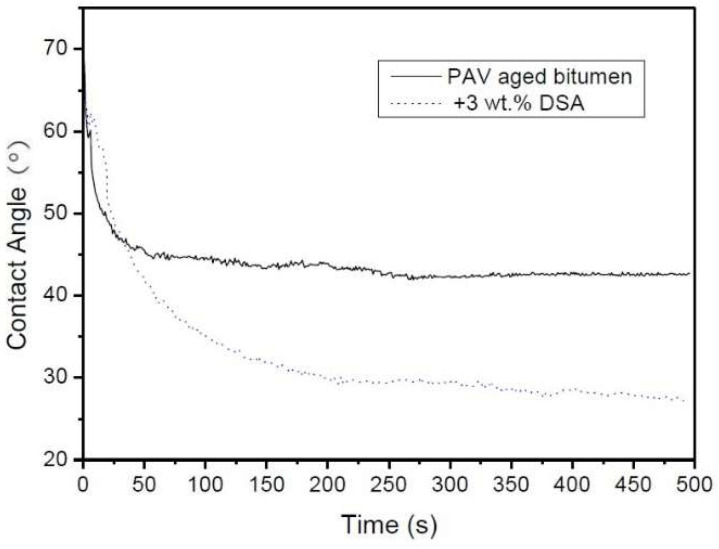
Evolution of contact angle with time during the spreading test, at 110 °C, for a PAV aged bitumen before and after DSA modification.

With this aim, Figure 13 presents the decrease of contact angle during the spreading test, at 110 °C, for a PAV-aged bitumen before and after DSA modification. In both cases, two different regimes can be clearly distinguished. On the one hand, the viscous regime, characterised by a quicker spreading rate, is observed at short times. On the other hand, at longer times, the so-called capillary-adhesive regime appears, where both samples reach an equilibrium configuration [20]. It is noteworthy that PAV-aged bitumen presents a fast spreading pace in the viscous regime, which precedes a quick flattening and equilibration of the curve at angles around 42°. By contrast, even though DSA-modified bitumen exhibits a slower spreading rate, especially at longer times, it finally reaches much lower values of contact angles on dried aggregate interfaces and, therefore, presents better wettability. In addition, DSA addition yields a near complete wetting of the aggregate surface with very low contact angles (26°), indicating a remarkable enhancement of the coating capacity after the modification.

This result can be related to improved physicochemical interactions developed at the bitumen–aggregate interface. Since greywacke is a siliceous-type mineral and considered an acidic one, its surface chemistry does not favour adhesion, which negatively acts on the bonding process in collaboration with the hindrance caused by the naphthenic acids of bitumen [36].

Hence, taking into account that reactions of DSA molecules with acid groups reducing the polarity of the resulting molecules due to the attachment of a non-polar tail, this process would favour binder–aggregate interactions, leading to improved adhesion.

**Figure 14:**
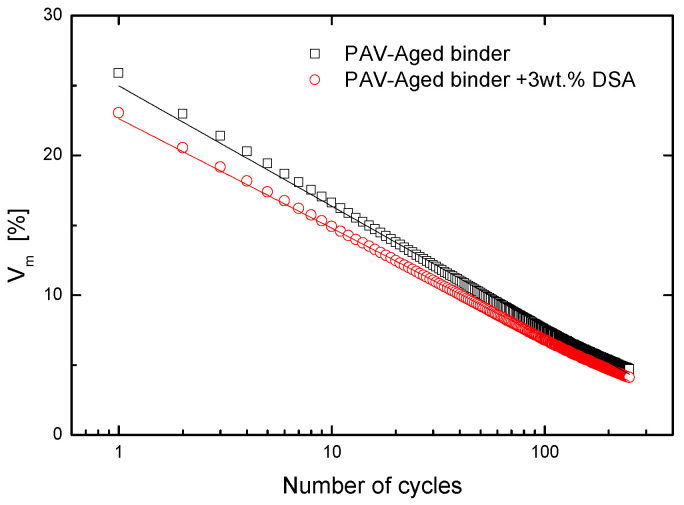
Evolution of air content with number of cycles for a PAV aged bitumen before and after DSA modification.

Finally, the workability and compactibility of the asphalt mixtures are assessed by the analysis of the densification curves of a model AC16S asphalt mixture. Figure 14 portrays the evolution of air void content for two mixtures formulated with a PAV-aged bitumen containing DSA and in absence of it. The results clearly point out that the addition of DSA led to a lower content of air voids in the mixture and, therefore, to a minor resistance to compaction for the same number of compaction cycles, confirming that the use of DSA enhances the workability and compactibility of the asphalt mixtures. This result can be attributed to the lower viscosity obtained after the modification, as well as to the enhanced wettability, further proving the potential rejuvenating capacity of the DSA [30,37].

## 4. Conclusions

As expected, RTFOT and PAV accelerated ageing in the laboratory, when applied to the selected bitumen, causing a remarkable hardening at both low and high temperatures. This well-known effect is mainly a consequence of the thermo-oxidative reactions that provoke a reduction in the aromatics content, accompanied by a rise in the concentration of resins and asphaltenes, especially after PAV. DSA and oil modification yields a notable softening on both neat and aged samples, as shown by the drop of the storage and loss moduli, viscosity at 60 °C and flexural BBR stiffness at low temperature. Whereas in the case of oil, the softening is due to a dilution effect; for DSA, the modification is related to the physicochemical processes, as the reactive surfactant tends to link chemically to polar bitumen compounds, generating steric hindrances that partially hamper the formation of large asphaltenic structures. In addition, the obtained results also indicate that DSA is a promising ageing inhibitor to bitumen.

In general, DSA-modified bitumen presents the greatest stiffening reduction, especially in the intermediate-high temperature range for neat and RTFOT-aged samples, although its softening capacity is slightly lessened after PAV testing. However, according to BBR results oil-modified bitumen seems to present a slightly better low-temperature performance.

During the ageing process, the rejuvenation effect is accompanied by the partial evaporation/degradation of DSA molecules, diminishing its effectiveness after PAV completion. Notwithstanding, results show evidence that, after ageing, a number of DSA molecules are still attached to polar bitumen compounds, hence affecting the colloidal stability, which leads to the reported bitumen softening.

Finally, dynamic contact angle measurements demonstrate that DSA addition yields a remarkable enhancement in the wettability of a siliceous-type aggregate, also offering a better compactibility of a model bituminous mixture.

## Figures and Tables

**Table 1:** Particle size distribution of AC16S and mixtures tested.

Sieve Size (mm)	AC16S	Sample
22.4	100	100
16	90–100	94.3
8	60–75	65.4
4	35–50	39.0
2	24–38	26.8
0.5	11–21	14.9
0.25	7–15	11.3
0.063	3–7	5.1

**Table 2:** Zero-shear limiting viscosities obtained from steady-state flow tests, at 60 °C; and Brookfield viscosities, at 135 °C, for unaged neat and modified bitumens.

Sample	Processing Conditions	η (60 °C) Pa·s	η (135 °C) Pa·s
Neat	-	205	0.414
3 wt.% oil	150 °C, 1 h	125	0.331
3 wt.% DSA	90 °C, 1 h	99.3	
	120 °C, 1 h	100	
	150 °C, 1 h	96.4	0.298
	180 °C, 1 h	102	
	150 °C, 2 h	114	
	150 °C, 3 h	122	

**Table 3:** Selected AASHTO parameters for neat and modified bitumens.

		AASHTO	B85/100	3 wt.% Oil	3 wt.% DSA
T when G*/sinδ = 1 kPa (°C)	original	T315	65.4	61.9	62
T when G*/sinδ = 2.2 kPa (°C)	RTFOT	T315	65.4	61.9	62.0
Weight loss (%)	RTFOT		0.39	0.38	0.98
T when G*·sinδ = 5 MPa (°C)	PAV	T315	20.1	15.8	18.7
T when S(t) = 300 MPa	PAV	T313	−17.9	−22.7	−19.3
S(t) (MPa) at −12 °C	PAV	T313	304	173	257
m-Value at −12 °C	PAV	T313	0.318	0.340	0.319

**Table 4:** Parameters C1 and C2 of WLF equation and ‘‘mechanical’’ glass transition temperature, T_g_^max(G″)^.

Bitumen	Sample	C_1_	C_2_	T_g_^max(G″)^ (°C)
B85/100	original	16.58	163.24	−16.6
	RTFOT	11.28	109.79	
	PAV	13.30	116.26	
3 wt.% oil	original	14.6	155.79	−21.9
	RTFOT	11.16	114.34	
	PAV	13.42	124.64	
3 wt.% DSA	original	12.91	142.65	−22.1
	RTFOT	10.08	103.23	
	PAV	13.53	123.28	

## Data Availability

The data presented in this study are available on request from the corresponding author.
